# Myotonic Dystrophy—A Progeroid Disease?

**DOI:** 10.3389/fneur.2018.00601

**Published:** 2018-07-25

**Authors:** Peter Meinke, Stefan Hintze, Sarah Limmer, Benedikt Schoser

**Affiliations:** Friedrich-Baur-Institute at the Department of Neurology, University Hospital, Ludwig-Maximilians-University Munich, Munich, Germany

**Keywords:** myotonic dystrophy, segmental progeroid disorder, nuclear envelope, premature aging, DNA repair

## Abstract

Myotonic dystrophies (DM) are slowly progressing multisystemic disorders caused by repeat expansions in the *DMPK* or *CNBP* genes. The multisystemic involvement in DM patients often reflects the appearance of accelerated aging. This is partly due to visible features such as cataracts, muscle weakness, and frontal baldness, but there are also less obvious features like cardiac arrhythmia, diabetes or hypogammaglobulinemia. These aging features suggest the hypothesis that DM could be a segmental progeroid disease. To identify the molecular cause of this characteristic appearance of accelerated aging we compare clinical features of DM to “typical” segmental progeroid disorders caused by mutations in DNA repair or nuclear envelope proteins. Furthermore, we characterize if this premature aging effect is also reflected on the cellular level in DM and investigate overlaps with “classical” progeroid disorders. To investigate the molecular similarities at the cellular level we use primary DM and control cell lines. This analysis reveals many similarities to progeroid syndromes linked to the nuclear envelope. Our comparison on both clinical and molecular levels argues for qualification of DM as a segmental progeroid disorder.

## Introduction

Myotonic dystrophies (DM) are slowly progressing multisystemic disorders characterized by myotonia, muscle weakness, cataracts, and cardiac arrhythmia that can evolve into cardiomyopathy, insulin insensitivity and diabetes, testicular failure, and hypogammaglobulinemia (1, 2). The spectrum of DMs includes two types: type 1 (DM1) and type 2 (DM2) which are caused by mutations in two different genes. The age of onset of DMs ranges from congenital forms at birth to late onset at ~70 years. Clinical symptoms cover muscular weakness, cataracts, balding, skin changes, and diabetes mellitus, often mirroring the appearance of accelerated aging. While the pathomechanism of DM has been shown to be a general splicing defect, it remains unclear what genes and splice variants yield particular pathologies in the affected tissues. Here, we provide a clinical description of aging symptoms in DM and compare this to “typical” progeroid disorders which mimic physiological aging and are caused by mutations in nuclear envelope (NE) proteins or DNA-repair proteins. Furthermore, we directly investigate some molecular hallmarks of aging in primary cell lines of DM patients.

## Myotonic dystrophy

Myotonic dystrophy (DM) can be caused by mutations in two genes: *DMPK* and *CNBP*. In both cases the disease is caused by an expansion of repeat elements within non-coding regions of the genes. Those repeats are transcribed and therefore contained within the pre-spliced mRNA. It is thought that RNA containing the expanded repeat forms hairpin structures and accumulates in foci in the nucleus. Several RNA-binding proteins are then recruited to these foci where they interact strongly with the mutant RNA. Among these proteins are MBNL proteins which are involved in alternative splicing (3). The accumulation of these proteins in the mRNA foci is thought to result in their depletion from the rest of the nucleus, resulting in turn in general mis-splicing and toxicity. This mis-splicing tends to revert the splicing pattern to comprise many embryonic splice variants. Phenotypically myotonic dystrophy is an autosomal dominant disease with predominant myotonia and muscle wasting. Furthermore eyes, heart, bone, skin, the endocrine system, gastrointestinal organs, and the central as well as the peripheral nervous system can be affected. The repeat length can vary between different tissues significantly (4). This complicates a prediction of the clinical development of patients as the repeat length is usually measured in DNA gained from blood.

DM1 causing mutations are an expansion of a CTG repeat in the 3' UTR of the *DMPK* gene (5). The general tendency is that the longer the expanded repeat the more severe the resulting phenotype is. Anticipation is commonly observed; the number of repeats typically increases in offspring over their parents so that the heritable disease tends to be of increasing generational severity. Up to 35 CTG triplets are considered normal, a repeat length between 35 and 49 is considered to be a premutation. Between 50 and ~150 repeats have been observed in a mild expression of the phenotype and ~100 to 1000 CTG repeats were identified in patients with classical DM. Repeats consisting of more than 1,000 CTG-triplets result in congenital DM, the most severe expression of the disease. The sexual inheritance also affects the severity of the disease: maternal inheritance results in more severe clinical features than paternal inheritance (6, 7).

Milder DM phenotypes can encompass cataracts, mild myotonia, or diabetes mellitus only, and the age of onset ranges between 20 and 70 years (8). Additional symptoms described in classical DM1 include distal muscle weakness, fatigue, cardiac conduction defects, neuropathy, endocrinopathies (on top of diabetes mellitus), and alopecia. Age of onset for the classical phenotype ranges between 10 and 30 years. In congenital DM1 affected children suffer severe and generalized weakness, hypotonia and respiratory problems after birth. One study further found that DM1 patients may have an increased risk of skin cancer (9).

DM2 mutations are located within intron 1 of the *CNBP* gene: more than 75 CCTG repeats have been described as disease causing (10). Unlike DM1 there is no described correlation between repeat length and disease severity in DM2. DM2 is considered a clinically more benign disorder than DM1 (11) and can be distinguished by a proximal muscular dystrophy and sparing of facial muscles (11), and the lack of a congenital form or the severe central nervous system involvement observed in DM1 (10, 12).

The fact that DM1 and DM2 are not clinically identical indicates that there are additional factors contributing to the disease pathomechanism besides the sequestration of splicing factors. In DM2 the repeat expansions tend to be longer than in DM1 so that one would expect DM2 to be more severe, but the opposite is the case: DM2 is clinically more benign. Thus it is possible that apart from the RNA toxicity the respective gene loci are contributing in different ways to the phenotype.

Due to its multisystemic involvement DM was suggested decades ago to be a segmental progeroid syndrome (13). Later it was also proposed as a model for premature muscle aging (14) and it is possible that in some mild cases DM might mimic sarcopenia (15, 16). Skin abnormalities frequently observed in DM1 and DM2 are also regarded as indicators for premature aging (17).

## Nuclear envelope linked progeroid syndromes

A group of progeroid disorders is caused by mutations in proteins of the nuclear envelope (NE) and also proteins involved in their processing. The NE is a double membrane system enclosing the genome in eukaryotic cells (18). Nuclear envelope transmembrane proteins (NETs) reside within the NE and it is underlaid by a meshwork of intermediate filament proteins, the nuclear lamina (19, 20). NE proteins lost or mutated in progeroid syndromes include lamin A and BAF. Apart from progeroid syndromes mutations in the lamin A encoding gene *LMNA* cause several tissue specific diseases (including muscular dystrophy, neuropathy and lipodystrophy). The progeroid syndromes caused by *LMNA* mutations encompass Hutchinson-Gilford progeria syndrome (HGPS), mandibuloacral dysplasia (MAD), Malouf syndrome, and several atypical progeroid syndromes that cannot be assigned clearly. Lamin A is an intermediate filament protein which undergoes post-translational processing for farnesylation. It has functions in mechanical stability, higher-order genome organization, chromatin regulation, transcription, DNA replication, and DNA repair (21). The major protein involved in its post-translational processing is the zinc metalloprotease STE24, encoded by the *ZMPSTE24* gene. Mutations in the *ZMPSTE24* gene cause mandibuloacral dysplasia (MAD) and restrictive dermopathy (RD). BAF (barrier to autointegration factor), a DNA binding protein, is encoded by the *BANF1* gene. Its functions include chromatin remodeling, gene expression, and DNA damage repair (22). It has been shown to interact with the LEM domain containing NETs emerin, MAN1 and Lap2β as well as with lamin A (23–25).

Hutchinson-Gilford progeria syndrome (HGPS) is most commonly caused by the *de novo* heterozygous *LMNA* mutation c.1824C>T; p.G608G which activates a cryptic splice site and causes the deletion of 50 amino acids. This deletion includes the cleavage site necessary for maturation of lamin A by post-translational processing (26, 27). Affected individuals appear healthy at birth, but develop a progeroid phenotype within 1–2 years. This comprises a short stature, low body weight, early loss of hair, loss of subcutaneous fat, localized scleroderma-like skin conditions, osteolysis, and facial features resembling aging (small face and jaw, prominent eyes, pinched nose, thin lips, and protruding ears). In most cases cardiovascular problems are the reason for death in the second decade of life (28, 29).

Mandibuloacral dysplasia (MAD) can be caused by recessive mutations in *LMNA* [MADA, (30)] or *ZMPSTE24* [MADB, (31)]. While the *LMNA* mutations tend to be homozygous or compound heterozygous missense mutations, *ZMPSTE24* mutations resulting in MADB tend to be a combination of missense and nonsense mutations (32). Patients are characterized by postnatal growth retardation, craniofacial anomalies with mandibular hypoplasia, skeletal malformations, osteolysis of distal phalanges, and clavicles, skin changes such as atrophy, and speckled hyperpigmentation, insulin resistance, and diabetes, and lipodystrophy which appears to be partial in MADA and generalized in MADB (32).

Restrictive dermopathy (RD) is caused by homozygous or compound heterozygous nonsense *ZMPSTE24* mutations resulting in a loss of the protein (33). The term RD describes a rare, lethal, genodermatosis. Affected children die before birth or within the first week of life. Clinical features include tightly adherent thin skin, prominent vessels, characteristic facial features (“O” shaped mouth), generalized joint contractures, dysplasia of clavicles and respiratory insufficiency (32, 34).

Malouf syndrome is caused by heterozygous *LMNA* mutations within the N-terminal parts of lamin A. In 2003 mutations were identified in patients originally described as suffering from Werner syndrome, but with no mutation in the *RECQL2* gene and therefore named atypical Werner syndrome (35)–though if the clinical phenotypes were actually matching Werner syndrome was not absolutely clear (36, 37). Later work (38) noted the phenotypic similarity to patients described by Malouf et al. (39). Described clinical findings include hypergonadotropic hypogonadism, cardiomyopathy, blepharoptosis, mild mental retardation, prominent nasal bones, scleroderma-like skin, and lipodystrophy (35, 38, 39).

In addition to these progeroid disorders there are several cases of so called atypical progeroid syndromes caused by *LMNA* mutations. These cases are often linked to a specific mutation and show overlaps between well described *LMNA* or *ZMPSTE24* linked diseases. Therefore it's not possible to assign them clearly to a syndrome (40–44).

Nestor-Guillermo progeria syndrome (NGPS) is caused by recessive mutations in the *BANF1* gene (45). Patients start to develop a failure to thrive at age of 2, the skin becomes dry and atrophic and they develop a generalized lipoatrophy, osteoporosis, and osteolysis.

Amongst these several NE-linked progeroid syndromes, the age of onset and life expectancy vary, but similarities include skin abnormalities (scleroderma-like, atrophy or speckled hyperpigmentation), osteolysis/osteoporosis, loss of hair, cardiac involvement, insulin resistance, typical facies, and in some cases muscular weakness. No noteworthy increased risk of cancer amongst these disorders has been reported (Table 1).

**Table 1:** Overview comparing myotonic dystrophies with selected nuclear envelope- and DNA repair- linked progeroid syndromes.

**Disease**	**Type**	**Gene**	**Age of onset**	**Age of death**	**Skin**	**Bones**	**Eyes**	**Muscle /Fat**	**Developmental delay**	**Neuro-degeneration**	**Diabetes mellitus**	**Hair**	**Cancer**
Myotonic Dystrophy type 1 (DM1)	Mild	*DMPK*	20–70 yrs	60 yrs–normal	dysplastic nevi, alopecia, xerosis and seborrheic dermatitis		cataracts	myotonia	no		diabetes mellitus	alopecia	possibly increased risk
	Classic		10–30 yrs	48–55 yrs	dysplastic nevi, alopecia, xerosis and seborrheic dermatitis		cataracts	myotonia, muscular dystrophy	no	axonal peripheral neuropathy, CNS involvment	diabetes mellitus	alopecia	possibly increased risk
	Congenital		Birth to 10 yrs	Neonatal / 45 yrs	dysplastic nevi, alopecia, xerosis and seborrheic dermatitis		cataracts	muscular dystrophy	yes	yes	diabetes mellitus		possibly increased risk
Myotonic Dystrophy type 2 (DM2)		*CNBP*	3rd decade	Sudden death due to cardiac involvement possible	dysplastic nevi, alopecia, xerosis and seborrheic dermatitis		cataracts	myotonia, muscular dystrophy	no	in a few cases	diabetes mellitus	rarely alopecia	possibly increased risk
Hutchinson-Gilford progeria syndrome (HGPS)		*LMNA*	1–2 yrs	14 yrs	scleroderma-like	osteolysis	no	partial lipodystrophy	yes	no	diabetes mellitus	loss	no increased risk
Mandibuloacral dysplasia (MAD)	MADA	*LMNA*	4-5 yrs	normal life expectancy	skin atrophy, calcinosis	osteolysis	no	partial lipodystrophy	yes	no	diabetes mellitus	alopecia	no increased risk
	MADB	*ZMPSTE24*	2 yrs		skin atrophy	osteolysis	no	generalized lipodystrophy	yes	no	diabetes mellitus	alopecia	no increased risk
Restrictive dermopathy (RD)		*ZMPSTE24*	Antenatal, Neonatal	mean age of 13.5 years	hyperkeratosis		hypertelorism, entropion	no	yes			absent/spare eyebrows, -lashes, lanugo	no increased risk
Malouf syndrome		*LMNA*	Infancy, neonatal	18-26 yrs	no	osteoporosis	ptosis	lipodystrophy	no	mental retardation (some patients)	no	no	no increased risk
Nestor-Guillermo progeria syndrome (NGPS)		*BANF1*	2 yrs	Third decade of life	dry, atrophic	osteoporosis, osteolysis	propotosis	generalized lipoatrophy	yes	no	no	loss	no increased risk
Werner syndrome		*WRN*	Median age 13 yrs	Median age of 54	scleroderma-like	osteoporosis	cataracts	Muscle atrophy	yes	brain atrophy in 40%	diabetes mellitus	loss, premature greying	increased risk
Bloom syndrome		*BLM*	birth	Median age 27	photosensitivity, pigmentation abnormalities	no	no	no	yes	mild retardation, learning disability (some patients)	non-insulin-dependent diabetes mellitus	hypertrichosis	increased risk
Cockayne syndrome (CS)	CSA	*ERCC8*	1 yr	12 yrs	photosensivity, wrikeled and premature aged		cataracts, pigmentary retinopathy	denervation myopathy	yes	intellectual disability	diabetes mellitus	thin, dry and premature greying	no increased risk
	CSB	*ERCC6*	birth	7 yrs						severe mental retardation			

### DNA repair linked progeroid syndromes

Another group of progeroid diseases are caused by mutations in DNA-repair proteins. Those encompass mutations in RecQ protein-like helicases (RECQL) and nuclear excision repair (NER) proteins. RecQ helicases play major roles in genome maintenance and stability (46). Mutations in genes encoding members of this protein family are causative for the premature aging disorders Werner syndrome and Bloom syndrome. NER proteins repair single stranded DNA damage—particular UV-induced DNA damage. Progeroid syndromes caused by mutations in NER protein encoding genes include Cockayne syndrome, Xeroderma pigmentosum, and Trichothiodystrophy.

Werner syndrome is caused by recessive mutations in the RECQL2 protein encoding *WRN* gene that result in a loss of protein by creating new stop codons or cause frameshifts resulting in a premature stop codon (47). RECQL2 is involved in DNA double-strand break repair where it regulates the pathway choice between classical and alternative non-homologous end joining (48) and relocalizes from the nucleolus to other nuclear regions upon DNA damage (49). It is suggested to be involved in telomere replication (50). Werner syndrome patients have scleroderma-like skin changes, cataracts, osteoporosis, arteriosclerosis, diabetes mellitus, cancer, characteristic “birdlike” facies, and can have alopecia (51).

Mutations in the *BLM* gene, encoding RECQL3, cause Bloom syndrome. The inheritance is recessive, and mutations result in a loss of protein or loss of function (52, 53). RECQL3 is involved in DNA replication and repair, where it acts in several steps during homologous recombination during DNA double-strand break repair (54). Patients present with pre- and postnatal growth deficiency, UV-sensitivity, hypo-, and hyperpigmented skin, and predisposition to malignancy (55).

Cockayne syndrome (CS) is distinguished into type A [CSA, caused by recessive *ERCC8* mutations (56)] and type B [CSB, caused by recessive *ERCC6* mutations (57)]. CSA patients show a progeroid appearance with slow growth and development, skin photosensitivity, thin and dry hair, pigmentary retinopathy, sensorineural hearing loss and dental caries (58). CSB patients are characterized by failure to thrive, severe mental retardation, congenital cataracts, loss of adipose tissue, joint contractures, distinctive face with small, deep-set eyes, and prominent nasal bridge, kyphosis, sensorineural hearing loss, and cachectic dwarfism (59).

Xeroderma pigmentosum (XP) is a rare autosomal recessive disorder with patients showing acute photosensitivity and a predisposition to skin cancer on sun-exposed areas of the body (60). XP is caused by mutations in the *XPA, ERCC3, XPC, ERCC2, DDB2, ERCC4, ERCC5*, and *POLH* genes. Another NER protein associated disease is Trichothiodystrophy (TTD). Patients display a wide variety of clinical features which includes cutaneous, neurologic and growth abnormalities as well as intellectual/developmental disabilities, ocular abnormalities and decreased fertility (61). Causative mutations have been described in the *ERCC3, GTF2H5, MPLKIP, GTF2E2, ERCC2*, and *RNF113A* genes.

In general these DNA repair linked progeroid disorders exhibit a frequent involvement of the skin (scleroderma-like, hyperpigmentation, increased photosensitivity), osteoporosis, and cataracts occur, and there is also frequently neuronal involvement. In addition this group of disorders tends to have an increased risk of cancer (Table 1).

## DM—accelerated aging at the molecular level?

Aging related defects can be observed at the cellular level in cells from patients with premature-aging disorders. Cellular hallmarks of aging include senescence, telomere attrition, genomic instability, mitochondrial dysfunction, and loss of proteostasis (62). There are observations of premature senescence in DM cells — cells obtained from distal muscle of congenital DM1 patients show a reduced proliferative capacity and an increased rate of telomere shortening. The reduced proliferative capacity observed in these cells was thought to be caused by a p16 dependent premature senescence (63, 64). DM2 myoblasts have also been shown to reach premature senescence, but in a p16 independent manner (65). Congenital DM1, but not DM2 myoblasts show differentiation defects (66, 67). There are also changes in epigenetic marks in both DM1 and DM2 patient cells, suggesting possible changes to genome organization: DM2 myoblasts exhibit heterochromatin accumulation (68) and the DM1 locus is methylated to varying degrees across the expanded repeats (69) and especially in congenital samples (70).

Another aging-associated feature is mitochondrial dysfunction. It is proposed that mitochondrial free radicals cause oxidative damage which is a driving force in cellular aging (71). Mutations in mitochondrial DNA (mtDNA) can lead to premature aging (72, 73). Increased mtDNA deletions have been reported in DM (74). Furthermore mis-regulation of the mitochondrial protein CoQ10 has been described in DM in generell (75) and EFTu, HSP60, GRP75 as well as Dienoyl-CoA-Isomerase specifically in DM2 (76). Another aging-linked feature is the loss of proteostasis. Proteostasis has been shown to collapse during aging (77) and there are indications that this occurs in DM1 and DM2: CTG repeat expressing mice activate the ubiquitin-proteasome pathway (78) and altered protein degradation has been shown in DM2 myotubes (76).

## Overlaps with typical progeroid disorders

DNA repair failure is certainly involved in the expansion of the repeats in both DM1 and DM2; this is potentially caused by slippage of DNA polymerase (79, 80). NER has also been shown to promote repeat expansion (81) and a polymorphism the MSH3 mismatch repair gene has been associated with somatic repeat instability (82). However, there are also reports indicating NE abnormalities in DM. In DM1 derived fibroblasts an altered localization of lamin A, lamin B1, and the NET emerin have been described (83). This altered localization includes distribution to invaginations of the NE, also known as nucleoplasmic reticuli (84). Knockdown of the zinc metalloprotease STE24, which is mutated in the progeroid syndromes MADB and RD and is a major player in the processing of prelamin A to mature lamin A, results in an enrichment of nucleoplasmic reticuli (85).

## Materials and methods

### Patient and controls

Primary human myoblast were obtained from the Muscle Tissue Culture Collection (MTCC) at the Friedrich-Baur-Institute (Department of Neurology, Ludwig-Maximilians-University, Munich, Germany). All control and patient materials were obtained with written informed consent of the donor. Ethical approval for this study was obtained from the ethical review committee at the Ludwig-Maximilians-University, Munich, Germany (reference 45-14). Repeat length was diagnosed on DNA extracted from blood. Age and sex of patients and controls are listed in Table 2.

**Table 2:** Primary myoblast cell lines used and characterization.

**Sample**	**Sex**	**Age at biopsy in years**	**Repeat length**	**Muscle of origin**	**Positive for Ki-67 staining in %**	**Positive for desmin staining in %**	**Used for immune-fluorescence**	**Used for western blot**
Ctrl-1	♂	43	–	M. biceps brachii	22.3	96.2	yes	yes
Ctrl-2	♀	36	–	M. biceps brachii	42.0	68.4	yes	no
Ctrl-3	♀	49	–	M. vastus lateralis	27.0	95.2	yes	yes
DM1-1	♂	38	200	unknown	n.d.	n.d.	yes	no
DM1-2	♂	34	240–430	M. deltoideus	16.6	70.1	yes	yes
DM1-3	♀	33	300–500	unknown	26.6	83.3	yes	yes
DM1-4	♂	27	400–600	unknown	n.d.	100	yes	no
DM1-5	♀	29	800–1500	unknown	37.9	50.3	yes	yes
DM2-1	♂	31	n.d.	unknown	57.6	48.0	yes	yes
DM2-2	♀	32	n.d.	M. vastus lateralis	43.0	86.0	yes	yes
DM2-3	♂	41	n.d.	M. rectus femoris	43.4	45.0	yes	yes
DM2-4	♀	37	n.d.	M. biceps brachii	n.d.	n.d.	yes	no
DM2-5	♂	35	n.d.	M. biceps brachii	20.0	94.4	yes	no

### Tissue culture

Myoblasts were grown in tissue culture using skeletal muscle cell growth medium (PeloBiotec, Munich, Germany). Cells were kept from reaching confluency to avoid differentiation. Passage numbers were matched for controls and patient cells for the respective experiments, throughout all experiments passage numbers 8 to 10 have been used. For differentiation DMEM containing 5% HS, was used. Myotubes were differentiated for 7 days. Cells were grown at 37°C in a 5% CO2 incubator.

### Immunohistochemistry

Myoblasts were fixed with methanol (−20°C). Following primary antibodies were used for staining: Ki-67 (Thermo Scientific, RM-9106-S0), emerin 5D10 and lamin A/C 4A7 (both provided by Glenn E. Morris). All secondary antibodies were Alexafluor conjugated and generated in donkey with minimal species cross-reactivity. DNA was visualized with DAPI (4,6-diamidino-2 phenylindole, dihydrochloride).

### Microscopy and image analysis

All images were obtained using an Olympus FluoView FV1000/BX 61microscope equipped with a 1.42 NA 60x objective and 3x zoom magnification. Image analysis was performed using ImageJ software. For quantification of nuclear invaginations at least 100 nuclei were counted for each measurement with the Olympus FluoView FV1000/BX 61 confocal microscope using the Z-drive to investigate the whole nucleus. For each sample at least two or three (depending on the fitness of each individual cell line) biological replicates were analyzed.

### Western blotting

Whole protein extracts were generated from myoblast cell cultures using an ultrasonic sonicator with a MS73 tip (Bandelin Sonopuls). The proteins were separated by SDS gel electrophoresis using 4–15% TGX gels (BioRad #456–8087). Western blotting was performed using the Trans-Blot^®;^ TurboTM system (BioRad). Proteins were transferred to low fluorescent PVDF membranes (part of Trans-Blot^®;^ TurboTM RTA Transfer Kit #170-4274). Membranes were blocked with 5% BSA or 5% skim milk in 1xTBS/0, 1% Tween^®;^ 20. Following primary antibodies were used: lamin A/C 4A7 (provided by Glenn E. Morris), lamin B1 (D4Q4Z, Cell Signaling) p16INK4A (ab108349, Abcam), p21 (Cell Signaling #2947). For quantification mouse antiGAPDH (Milipore #MAB374) or rabbit antiGAPDH (Cell Signaling GAPDH (D16H11) XP #5174) were used. As secondary antibodies we used donkey anti-mouse IRDye 680RD, donkey anti-mouse IRDye 800CW, donkey anti-rabbit IRDye 680RD and donkey anti-rabbit IRDye 800 CW. All western blot images were obtained using a Licor FC. Quantification was done using the Licor ImageStudio Software. Western blots were repeated at least two times to confirm the results.

## Results

**Figure 1:**
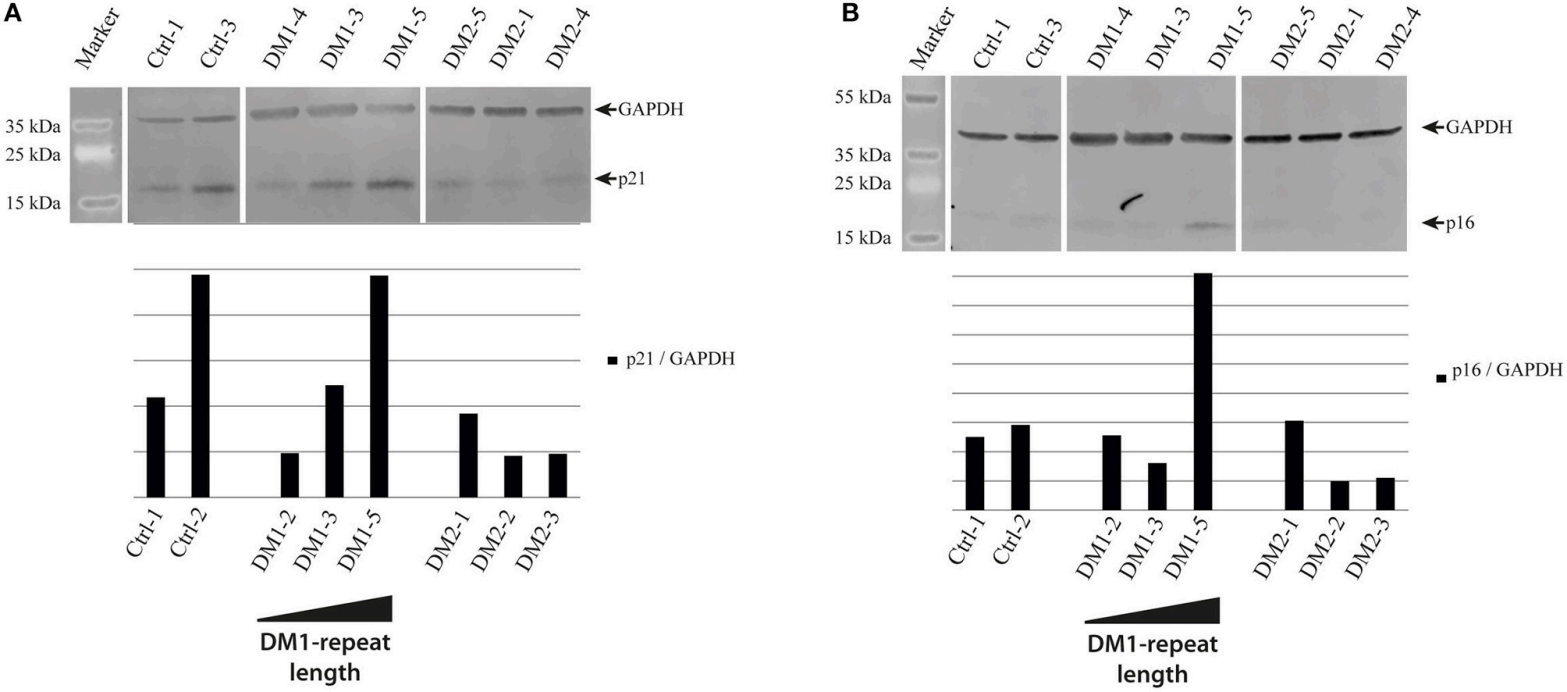
Cell cycle regulatory proteins in myotonic dystrophy. Western Blot and quantification of primary control, DM1 and DM2 myoblasts for cell cycle regulatory proteins p21 **(A)** and p16 **(B)**. DM1 samples are ordered according their diagnosed repeat length from left (small repeat) to right (long repeat).

**Figure 2:**
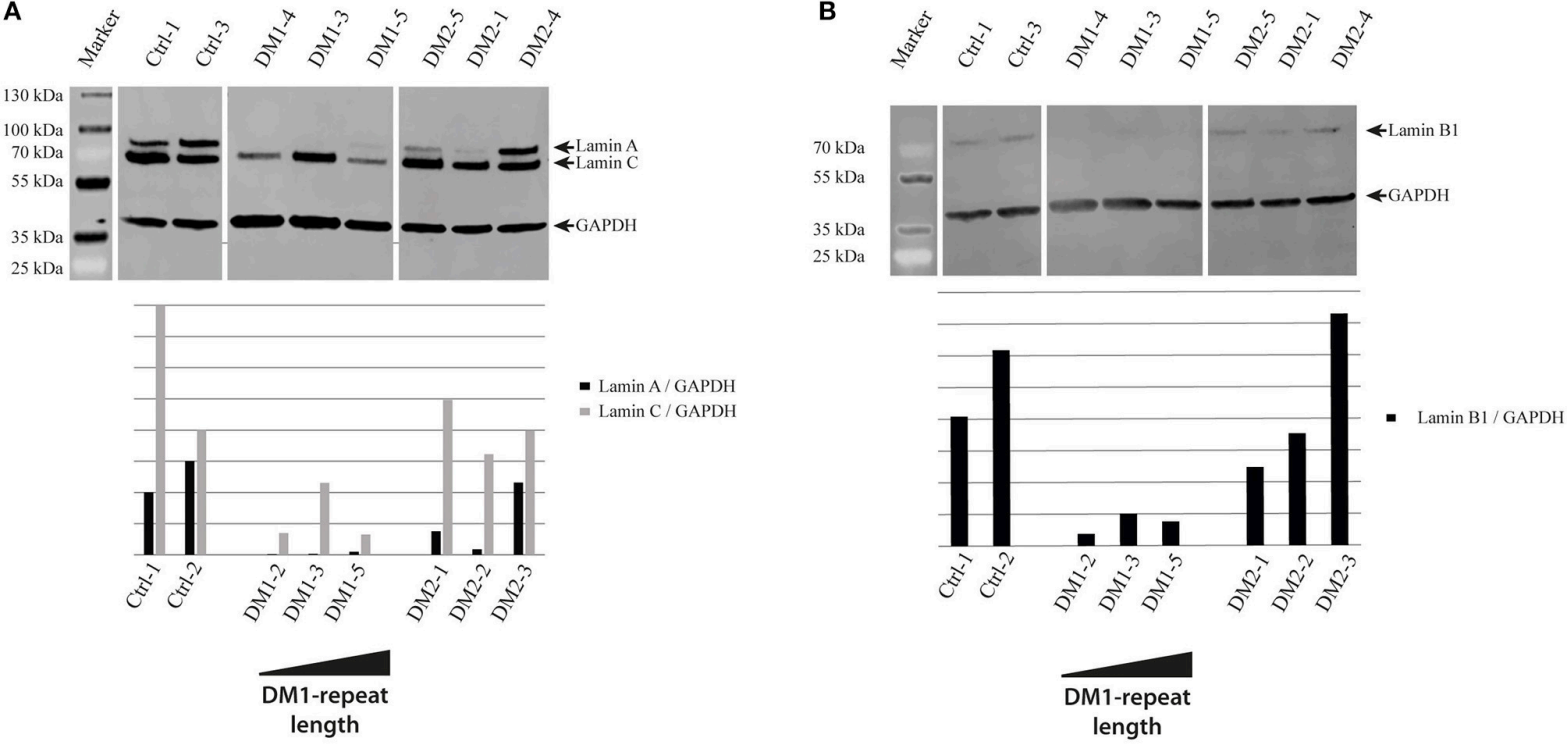
Lamina proteins in myotonic dystrophy. Western Blot and quantification of primary control, DM1 and DM2 myoblasts for lamin A and lamin C **(A)** and lamin B1 **(B)**. DM1 samples are ordered according their diagnosed repeat length from left (small repeat) to right (long repeat).

As cellular senescence is a hallmark of aging we decided to initially analyze cell cycle proteins linked to senescence in our primary human myoblast cell lines. First, we used Western blot analysis to quantify the expression of p21 and p16 (Figure 1). P21 is an inhibitor of the cell cycle (86), which fails to be up-regulated in DM2 myoblasts during differentiation (87). Western blot analysis of our myoblast cell lines shows only small changes in p21 expression (Figure 1A), however this may be within the observed expression level variations in primary myoblasts. P16 is a tumor suppressor protein that has previously been shown to be mis-regulated in congenital DM1 samples (63). There were no changes in p16 expression except in the DM1 cell line with the longest repeat where the expression was elevated. The next step was to quantify lamin B1, another senescence associated biomarker (88) which has not been investigated in DM before. While we do not see lamin B1 changes in DM2 myoblasts, it was down-regulated in all DM1 myoblasts tested (Figure 2B).

**Figure 3:**
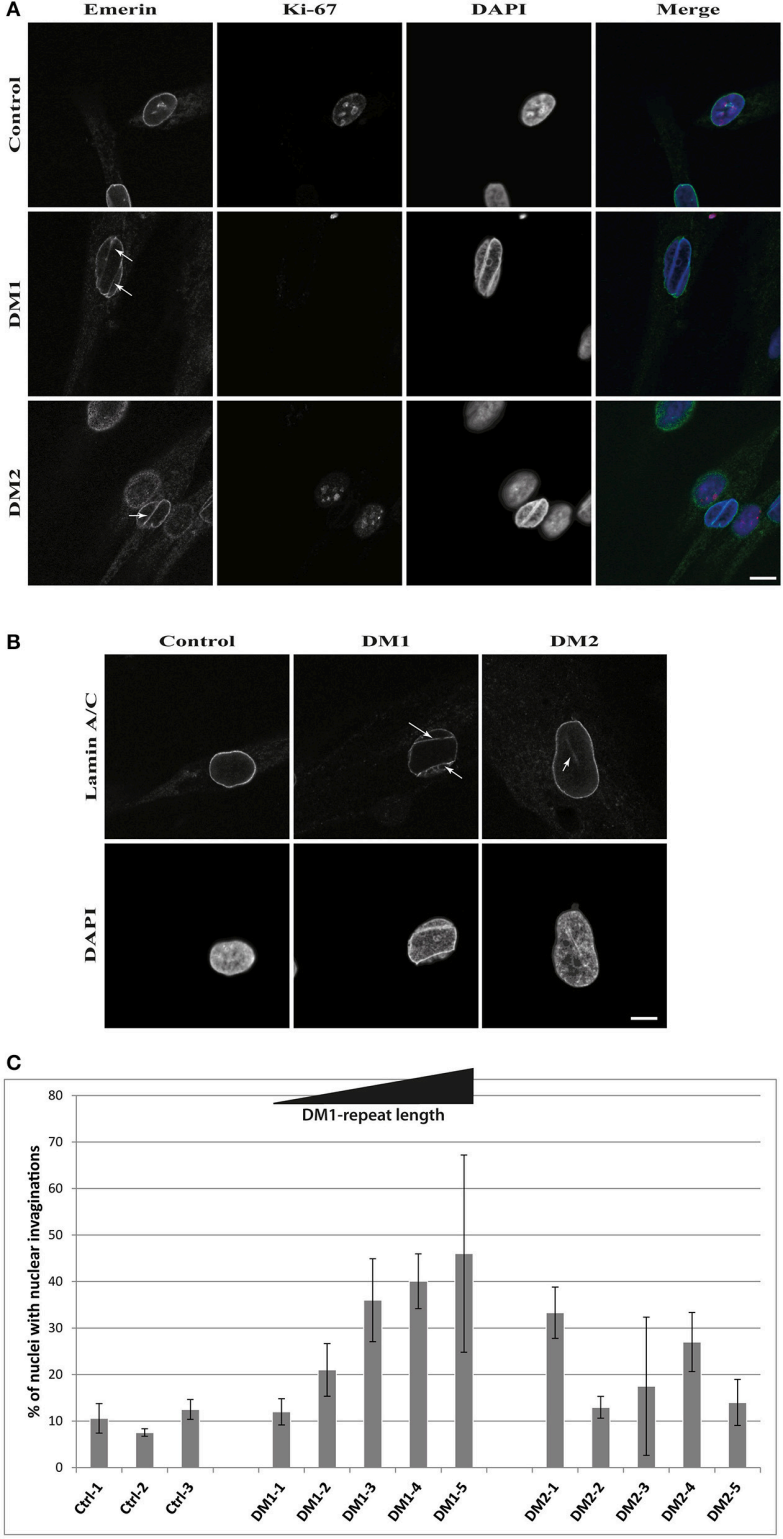
Nuclear envelope invaginations in myotonic dystrophy. Immunofluorescence staining of primary control, DM1 and DM2 myoblasts for **(A)** emerin and Ki-67 showing nuclear envelope invagination in DM1 and DM2 myoblasts, **(B)** confirmation of nuclear envelope invaginations by lamin A/C staining and **(C)** quantification of these structures in DM and control cell lines—standard deviation is shown. White arrows indicate invaginations of the nuclear envelope. Scale bar 10 μm.

**Figure 4:**
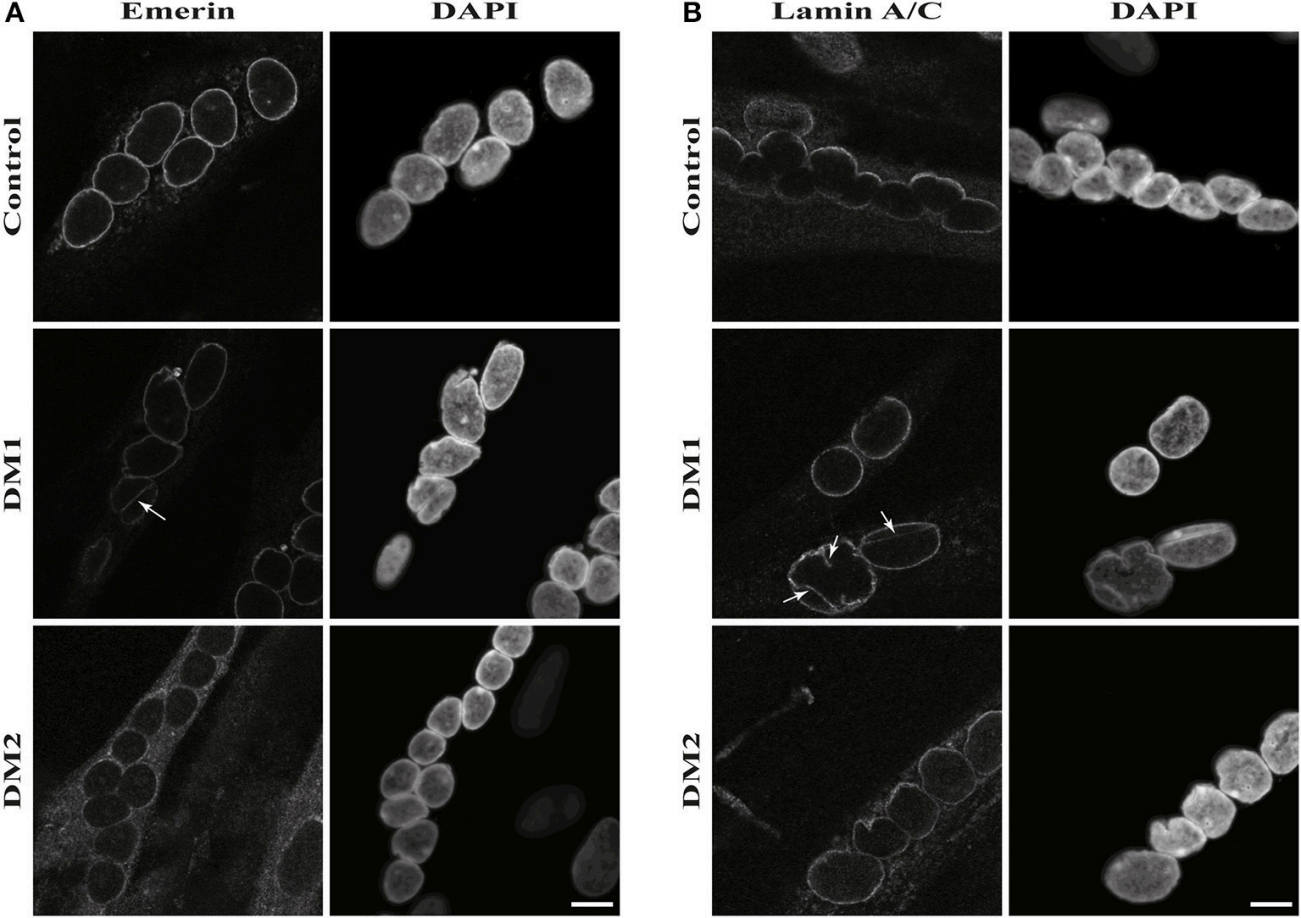
Nuclear envelope invaginations in myotonic dystrophy and control myotubes. Immunofluorescence staining of primary control, DM1 and DM2 myotubes for **(A)** emerin and **(B)** lamin A/C showing nuclear envelope invagination in DM1 myotubes. White arrows indicate invaginations of the nuclear envelope. Scale bar 10 μm.

The results of the lamin B1 quantification led us to further investigate effects on NE proteins. For this we quantified the expression of the other lamin subtypes A and C, finding that lamin A is strongly down-regulated in DM1 (Figure 2A). Immunofluorescence staining for lamin A/C shows an increased number of nuclei with invaginations in DM1 and DM2 myoblasts (Figure 3B). This has been confirmed and quantified by staining with the NET emerin (Figures 3A,C). For DM1, primary patient cell lines with longer CTG repeats show a greater percentage of nuclei with nuclear invaginations (Figure 3C). Co-staining with the proliferation marker Ki-67 revealed that all cells with nuclear invaginations are negative for Ki-67 and hence senescent. Staining of differentiated myotubes with lamin A/C and emerin shows the presence of nuclear invaginations in DM1 myotubes (Figures 4A,B).

## Discussion

The differences in cell cycle control described in DM1 and DM2 myoblasts gave rise to the idea that there are different pathomechanisms in each DM type. While for DM2 a down-regulation of the cell cycle inhibitor p21 has been shown (87), there is a reported mis-regulation of p16 in congenital DM1 (63). We can confirm both effects in our DM patient myoblasts, although the effects observed by us are not very strong and the mis-regulation of p16 in DM1 seems to be restricted to longer repeats (Figure 1).

Looking for a better senescence marker we quantified lamin B1, a protein of the NE that has been shown to be down-regulated in HGPS (89), cellular senescence (90) and normal aging (91). The down-regulation of lamin B1 occurs specifically in DM1 myoblasts and is independent of the repeat length (Figure 2B). This indicates that it could be a more relevant marker for DM1 in general than p16. Furthermore, the strong down-regulation of lamin A (Figure 2A) confirms that there are strong effects on the expression of nuclear lamina proteins in DM1. The composition of the nuclear lamina is important to achieve its multiple functions including mechanical stability, chromatin organization, transcriptional regulation, and response to oxidative stress (92, 93)–and the down-regulation of the lamins A and B1 is potentially having an effect on all these functions thus contributing to the phenotype.

Further evidence of NE involvement in DM comes from the enrichment of nuclear envelope invaginations in DM myoblasts (Figures 3, 4). DM myoblasts show more nuclei with these structures, with the strongest effects being observed in DM1. Moreover, all nuclei with invaginations have exited the cell cycle. In contrast to the lamin B1 levels, there appears to be a correlation of repeat length in DM1 and the percentage of nuclei positive for NE invaginations. The longer the diagnosed DM1 repeat the more myoblast nuclei in primary cell lines gained from those patients have invaginations of the nuclear envelope. This could be another overlap to NE-linked progeroid syndromes: knockdown of ZMPSTE24 also results in an enrichment of NE invaginations (85) and the loss of ZMPSTE24 in human results in RD, the most severe NE-linked progeroid syndrome (32). A possible explanation of how this contributes to the disease pathology is that mis-regulation of lamina proteins results in NE aberrations which force the cell to exit the cell cycle and enter senescence. Consequently, this might deplete the pool of myoblasts during muscle regeneration and contribute to the muscular dystrophy in DM via failed regeneration.

**Figure 5:**
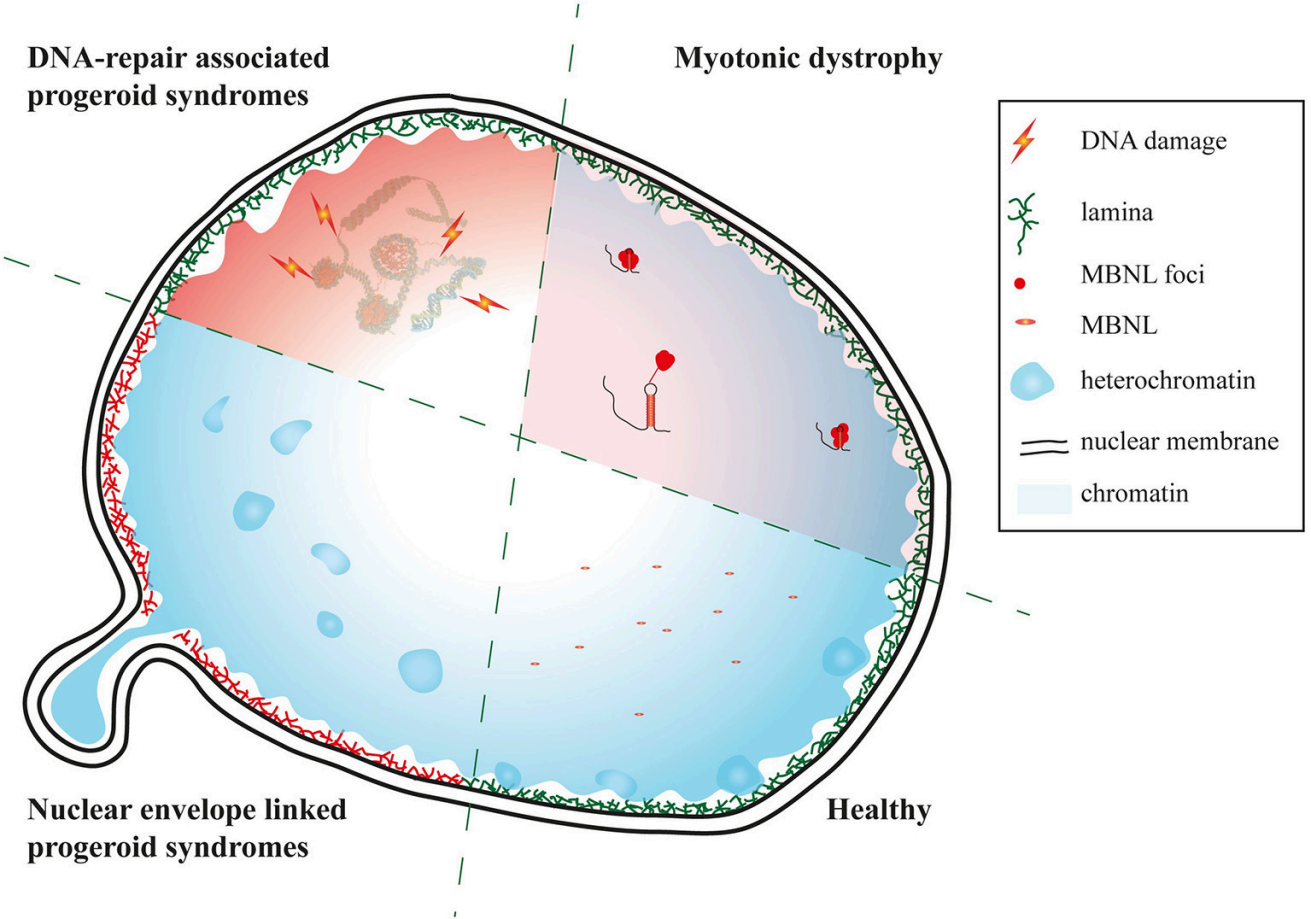
Myotonic dystrophy as a facet of progeroid syndromes? Schematic of affected nuclear regions and pathways in DM and nuclear envelope as well as DNA repair linked progeroid syndromes.

Taken together our results suggest that there is on both the clinical and molecular level clear evidence that DM reflects facets of segmental progeroid disorders (Figure 5).

## Conclusion

DMs qualify by clinical phenotypes as well as molecular features as segmental progeroid syndromes. In DM1 the composition of the NE is altered and there is an enrichment of nuclear invaginations likely contributing to the phenotype. As several NE-linked syndromes have muscle involvement, this further suggests the possibility of an overlap between NE-linked progseroid syndromes and DM1.

## Author contributions

PM and BS contributed to the conception and design of the experiments. PM wrote the manuscript, SH and SL performed the experiments.

### Conflict of interest statement

The authors declare that the research was conducted in the absence of any commercial or financial relationships that could be construed as a potential conflict of interest.
